# Combining Hi-C data with phylogenetic correlation to predict the target genes of distal regulatory elements in human genome

**DOI:** 10.1093/nar/gkt785

**Published:** 2013-09-03

**Authors:** Yulan Lu, Yuanpeng Zhou, Weidong Tian

**Affiliations:** State Key Laboratory of Genetic Engineering, Department of Biostatistics and Computational Biology, School of Life Science, Fudan University, Shanghai 200433, China

## Abstract

Defining the target genes of distal regulatory elements (DREs), such as enhancer, repressors and insulators, is a challenging task. The recently developed Hi-C technology is designed to capture chromosome conformation structure by high-throughput sequencing, and can be potentially used to determine the target genes of DREs. However, Hi-C data are noisy, making it difficult to directly use Hi-C data to identify DRE–target gene relationships. In this study, we show that DREs–gene pairs that are confirmed by Hi-C data are strongly phylogenetic correlated, and have thus developed a method that combines Hi-C read counts with phylogenetic correlation to predict long-range DRE–target gene relationships. Analysis of predicted DRE–target gene pairs shows that genes regulated by large number of DREs tend to have essential functions, and genes regulated by the same DREs tend to be functionally related and co-expressed. In addition, we show with a couple of examples that the predicted target genes of DREs can help explain the causal roles of disease-associated single-nucleotide polymorphisms located in the DREs. As such, these predictions will be of importance not only for our understanding of the function of DREs but also for elucidating the causal roles of disease-associated noncoding single-nucleotide polymorphisms.

## INTRODUCTION

Distal regulatory elements (DREs), including enhancer, insulator and repressor, are *cis*-regulatory elements that regulate gene expression from long distances. Though the precise mechanism of how DREs regulate target gene expression is not well understood, one widely held model of enhancer function proposes that on transcription factor (TF) binding and mediation by cohesin and mediators, enhancer can be brought proximately to the promoter of target genes through the bending of DNA structure, a process called DNA looping (1), which facilitates the regulation of target gene expression. Enhancers play central roles in many cellular processes, such as regulation of transcription, site-specific recombination and replication (2). For example, the expression of *NANOG* (3) and *OCT4* (4) that play important roles in human embryonic stem cell development are controlled by enhancers; members of the bone morphogenetic proteins (BMPs) family, such as *Bmp2*, *Bmp4*, *Bmp5* and *Gdf6*, which play important roles in developmental processes, are regulated by enhancers (5). Owing to its important functional roles, mutations on enhancers may disturb normal cell activities and cause diseases, such as aniridia (6), Hirschsprung’s disease (7), preaxial polydactyly (8) and X-linked deafness (9). A recent survey found that almost half of single-nucleotide polymorphisms (SNPs) that show significant association with common/complex diseases are located in noncoding regions that potentially are DREs (10), further highlighting the important roles of DREs.

To define the function of DREs, it is necessary to determine their target genes, such that the biological processes regulated by DREs can be inferred. A common way to determine the DREs that regulate a given gene of interest is to scan for conserved noncoding sequences that are located near the target gene in genome. For example, in *Fugu rubripes*, the enhancers of *Hoxb-1* and *Hoxb-4* were identified by searching for conserved noncoding sequence blocks upstream of the two genes through sequence comparisons with mouse genome (11); the enhancer of *Gli3* was also identified in a similar way (12). However, DREs can regulate target genes from a long distance (13), or even across chromosomes (14), and their relationships with the target genes are not limited to a one-to-one relationship (15). All these complicate the task of defining the target genes of DREs. Mark *et al.* (16) developed the Site Clustering Over Random Expectation (SCORE) algorithm to predict enhancer–target gene pairs that share common Transcription Factor (TF) binding sites, which does not require enhancer to be located close to target genes. However, sharing common TF binding sites is not required for enhancer–target gene interactions (17), limiting its usefulness in detecting enhancer–target gene relationships. Recently, Chromosome Conformation Capture (3C) (18) and Hi-C (19) as well as ChIA-PET (20) techniques have been developed to capture long-range chromatin interactions. Because enhancer and the target gene may form a chromosome looping structure during the interaction, the data generated by these techniques can be potentially used to identify DREs–target gene relationships. However, these data have not been explored for this purpose yet.

Hi-C experiment generates millions of short sequence reads. Each sequence read contain sequence fragments from two different chromosome locations resulted from a chromosome interaction event. If a Hi-C read can be mapped to both a DRE and a gene, then it is likely the gene is the target gene of the DRE. This makes Hi-C data useful for detecting DRE–target gene relationships. However, on one hand, the sequence data produced by Hi-C are noisy. On the other hand, Hi-C may capture random chromosome interactions as well. Thus, the direct use of Hi-C data to determine the target genes of DREs may generate many false positives. Given that a DRE regulates its target gene expression by forming a looping structure, we hypothesize that such looping structure may place strong evolutionary constraints on the evolution of both the DRE and the target gene. If for some reasons, either the DRE or its target gene is lost during evolution, then the evolutionary constraints placed by chromosome looping will disappear, which may result in the loss of its counterpart as well. With a large number of evolutionarily related genomes available, we should then be able to observe a positive correlation between the presence of a DRE and its target gene across the genomes, making it possible to use phylogenetic correlation to select DRE–target gene pairs from Hi-C data.

To test our hypothesis, in this study we first use DNase I hypersensitive sites (DHS) downloaded from UCSC genome browser to annotate DREs in human genome, as DREs are often located within open chromatin regions that are sensitive to DNase I digestion (21–23). Then, we download Hi-C data generated by a recent study (24), and annotate DRE–gene pairs using Hi-C reads. Next, by preparing phylogenetic profiles for each DRE and coding gene in the human genome using the pairwise genome alignments between human and 45 vertebrates, we investigate the phylogenetic correlation between DREs and genes annotated by Hi-C reads. We show that compared with random DRE–gene pairs, the Hi-C annotated DRE–gene pairs tend to be significantly phylogenetic correlated, and the correlation is stronger for DRE–gene pairs confirmed with more number of Hi-C reads or for those confirmed by Hi-C reads from more cell lines. Thus, we have developed a method that combines Hi-C read counts with phylogenetic correlation to predict DRE–target gene relationships. Analysis of the predicted DRE–target gene pairs reveals that genes related to the same DREs tend to be functionally related and co-expressed. In addition, we have shown with examples that the predicted DRE–target gene pairs can be used to infer the function of unknown genes, and to explain the causal roles of disease-associated SNPs located within DREs. As such, the predicted DRE–target gene pairs will greatly facilitate our understanding of the functional roles of DREs.

## MATERIALS AND METHODS

### Data collection and preprocessing

DHS annotations are downloaded from the wgEncodeRegDnaseClustered track (25) of University of California, Santa Cruz (UCSC) genome browser (26). The protein-coding gene annotations are the union of the Ensembl annotation (versions 64, genome version hg19) (27) and UCSC ‘knownGene’ annotation (26). A DRE is defined as a DHS that is not within any coding gene region and whose distance to the nearest Transcription Start Site (TSS) is longer than 2000 bp. Hi-C data are downloaded from the Gene Expression Omnibus (GEO) database (accession number GSE35156) (24), and include sequencing data of the two replicates of two cell lines (H1 hESC and IMR90). The ChIP-seq data of all histone modifications used in this study and EP300 are download from the ENCODE project (25) and the Roadmap Epigenomics (28), and then processed following the method in (29). For peak calling, we use MACS software with default parameters (30). The pairwise genome alignment data between human and other 45 vertebrate species, from lamprey to chimp, are downloaded from UCSC genome browser (26). The list of the 45 species can be found in the Supplementary Table S1.

### Generation of phylogenetic profiles and calculation of the correlations

See Figure 2 for the flowchart of phylogenetic profile preparation. Based on the pairwise genome alignments between human and the other 45 species, we collect all human sequence fragments that have one or more homologous sequences in any of the other 45 genomes. We identify the starting and ending positions of those fragments in human genome, and use those positions to divide the human genome into consecutive nonoverlapping sequence bins. For each sequence bin, we prepare a phylogenetic profile that consists of 0 or 1 with a length of 45; here, 1 indicates this sequence bin has a homologous sequence in corresponding specie and 0 otherwise. The phylogenetic profile of a DRE or a gene is then computed as the weighted average of the phylogenetic profiles of all sequence bins inside the corresponding sequence region by the bin length. The DRE sequence region is defined as the peak region of the DHS, while the gene region is defined as the sequence region from −1000 bp before TSS to gene end. Then, for each combination of DRE and gene, we calculate the Pearson correlation coefficient (PCC) using their phylogenetic profiles.

### Functional analysis

Gene Ontology (GO) enrichment analysis is performed using FuncAssociate (31). GO annotations with ‘Inferred from Electronic Annotation (IEA)’ evidence code are removed, and only GO terms with three or more annotated genes are used for enrichment analysis. GO terms with the adjusted *P* < 0.05 are considered significantly enriched. Functional similarity between two genes is computed using the Resnik functional similarity measure that considers not only the number of shared GO terms between two genes but also the specificity of the GO terms. Details about Resnik similarity measure can be found in (32). Resnik similarity score is computed using only GO terms belonging to the major GO branch of biological process. For co-expression analysis, we download the expression profiles of 79 human tissues from UCSC genome browser [track gnfAltas (33)]. For every two genes, we compute the PCC using their own expression profile across the 79 tissues.

## RESULTS

### Annotation of candidate DRE–target gene pairs using Hi-C data

We annotate protein-coding genes using a combined set of Ensembl and UCSC gene annotation (26,27), and obtain 22 685 genes. Putative regulatory elements are annotated based on the experimentally determined DHS downloaded from UCSC genome browser. We define DREs as those DHS that are not located within or close to any coding gene regions (see ‘Materials and Methods’ section for details), which results in 455 477 DREs. To identify DRE–target gene relationships from Hi-C data, we download the Hi-C data from Dixon *et al.* (24), which includes ∼1.2 billion sequence reads from two cell lines (H1 hESC and IMR90, both cell line have two replicas). We consider a DRE–gene pair as a candidate DRE–target gene pair if a Hi-C read contains the sequence fragments from both the DRE and the gene.

**Figure 1.**
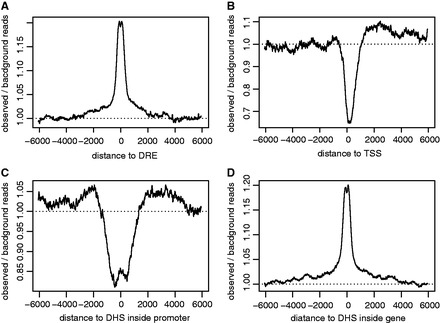
The distribution of Hi-C reads. The distribution of Hi-C reads around DREs (**A**), TSS (**B**), DHS inside promoter (**C**) and DHS inside gene (**D)**. For every target (e.g., a DHS or a DRE), the background Hi-C reads are the total number of Hi-C reads mapped to regions distant to the target (−5∼−10 kb and +5∼+10 kb to the center of the target) divided by the length of the regions (10kb). The observed Hi-C reads at a given nucleotide position are the averaged number of Hi-C reads mapped to that position with a window size of 50bp. The observed/background reads is then computed at every nucleotide position and averaged across all targets.

However, a Hi-C read may be mapped to different locations in a gene region, making it necessary to investigate which gene region should be used as the candidate region for Hi-C reads mapping. According to the current model of enhancer regulation, a DRE forms a looping structure with the promoter of its target gene. It seems natural to use the promoter region for Hi-C reads mapping. However, though the DRE regions are significantly enriched with Hi-C reads (Figure 1A), we find the promoter regions are significantly depleted with Hi-C reads (Figure 1B). We further inspect the distribution of Hi-C reads in DHS inside the promoter, and find that Hi-C reads are only locally enriched (Figure 1C). It is possible that a DRE may form the looping structure with the DHS inside the promoter region, but this looping structure may be transient, and will be unfolded upon RNA Pol II binding to the promoter. Indeed, we find Hi-C reads are significantly depleted around the center of RNA Pol II binding region (Supplementary Figure S1). The depletion of Hi-C reads in promoter suggests that it is not proper to use promoter region for Hi-C reads mapping. We then inspect the distribution of Hi-C reads along the gene region. We find that Hi-C reads are significantly enriched in DHS inside gene region (intragenic DHS) (Figure 1D), and ∼55% of Hi-C reads mapped to the gene region are located within the intragenic DHS region (±1000 bp from the center of the DHS) (data not shown). This suggests that a DRE may form a looping structure with its target gene not only at transcription initiation but also during transcription elongation. Thus, here we use the intragenic DHS for Hi-C reads mapping.

For each DRE–gene pair, we count the number of Hi-C reads that are mapped to both the DRE and the intragenic DHS. Intuitively, if a DRE–gene pair is confirmed by a higher number of Hi-C reads, it is more likely a true DRE–target gene pair. However, as genes with longer length tend to have more intragenic DHS, the sum of Hi-C reads over intragenic DHS is strongly biased by gene length (Supplementary Figure S2). To solve this problem, here we consider only the intragenic DHS that has the highest number of Hi-C reads among all intragenic DHS, and use the corresponding Hi-C read counts to represent the Hi-C read counts associated with the DRE–gene pair. Finally, we obtain 35 337 425 Hi-C annotated DRE–gene pairs, and consider them as candidate DRE–target gene pairs. However, only 4.5% of them are present in both cell lines (Supplementary Figure S3A), suggesting that a large fraction of them may be false positives.

### Combining Hi-C read counts with phylogenetic correlation to predict DRE–target gene pairs

To identify DRE–target gene pairs from the candidate pairs, an initial thought would be to rank those candidate pairs by Hi-C read counts, and then select those above a certain count. However, on one hand, Hi-C data are noisy. On the other hand, Hi-C may capture random chromosome interaction as well. Thus, selection based only on Hi-C read counts might still result in many false positives. As chromosome looping is a necessary step for a DRE to regulate its target gene expression, we hypothesize that such looping structure may place strong evolutionary pressure on the evolution of both the DRE and the target gene. As such, we would expect to observe a positive correlation between the presence of DRE and its target gene across evolutionarily related genomes, which may then be used for selecting DRE–target gene pairs additional to Hi-C read counts. However, there are currently not many known DRE–target gene pairs available for direct assessment of our hypothesis. Considering Hi-C annotated DRE–gene pairs are candidate DRE–target gene pairs, here we use them to indirectly inspect the phylogenetic correlation between DRE and target genes.

**Figure 2.**
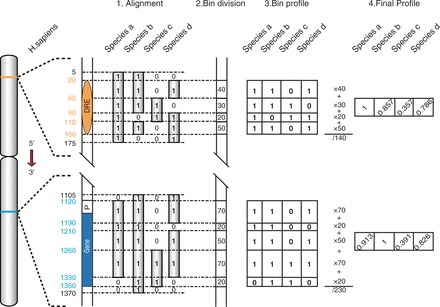
Flowchart of phylogenetic profile preparation. A hypothetical genome with a DRE and a gene and four other related species are used here to illustrate the process of phylogenetic profile preparation (see ‘Materials and Methods’ section for details).

**Figure 3.**
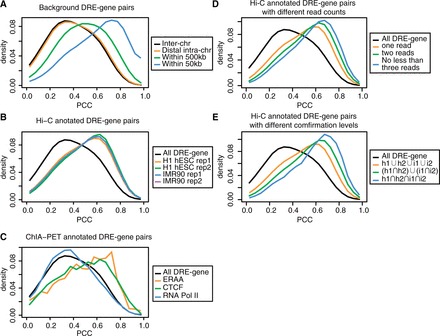
The distribution of the PCC of DRE–gene pairs. (**A**) The distribution of the PCC of DRE–gene pairs with different distance cutoffs: inter-chr, different chromosomes; distal intra-chr, the same chromosome but >500 kb; within 500 kb, <500 kb but >50 kb; within 50 kb, <50 kb. (**B**) The distributions of the PCC of Hi-C annotated distal DRE–gene pairs from the four data sets (the two replicas of the two cell lines). (**C**) The distribution of the PCC of ChIA-PET annotated DRE–gene pairs. (**D**) The distribution of the PCC of DRE–gene pairs with different Hi-C read counts. (**E**) The distribution of PCC of DRE–gene pairs with different confirmation level. h1 and h2 refer to the two replicas of H1 hESC, and i1 and i2 refer to the two replicas of IMR90, respectively. The background in B–E refers to distal DRE–gene pairs that are either from different chromosomes or with a distance >500 kb. DRE–gene pairs with negative PCC only account for a small fraction, and are not shown in the figures.

For a given DRE and a gene, we first prepare a phylogenetic profile for the DRE and the gene separately based on the pairwise genome alignment between human and 45 other vertebrate species; then, we calculate the PCC using the two profiles (Figure 2 and see ‘Materials and Methods’ section for details). A higher PCC indicates a stronger phylogenetic correlation. However, the PCC is strongly biased by the distance between the DRE and the gene in genome, with DRE and gene located closer to each other tend to have higher PCC (Figure 3A). For example, for DRE–gene pairs whose distances are within 50 kb and those within 50–500 kb, the mode PCC is 0.72 and 0.50, respectively. In contrast, for DRE–gene pairs whose distance is >500 kb, the distribution of their PCC is close to that of the DRE–gene pairs from different chromosomes (both the mode PCC are 0.35), indicating the bias caused by distance can be ignored. Therefore, in this study we focus only on distal DRE–gene pairs whose distance is >500 kb or who are from different chromosomes, and exclude the DRE–gene pairs that are within 500 kb to each other in the following analysis.

The Hi-C annotated distal DRE–gene pairs are strongly phylogenetic correlated: the mode PCC is 0.60, in contrast to 0.35 for background distal DRE–gene pairs (Figure 3B). Besides Hi-C technology, ChIA-PET that detects genome-wide chromatin interactions associated with certain proteins (20) can also be used to annotate DRE–gene pairs. Here, we analyze the PCC of ChIA-PET annotated distal DRE–gene pairs. We find that distal DRE–gene pairs annotated by ChIA-PET data based on CTCF (34) and ERRA (20) antibody are strongly phylogenetic related (Figure 3C). Interestingly, however, distal DRE–gene pairs annotated by ChIA-PET data based on RNA Pol II antibody (35) are not phylogenetic correlated, which seems consistent with the finding that Hi-C reads are depleted around RNA Pol II regions. Thus, it can be inferred from the above results that DRE and its target genes are strongly phylogenetic correlated. Because there are only a small number of ChIA-PET annotated DRE–gene pairs, here we only focus on Hi-C annotated DRE–gene pairs. Furthermore, we find that when a DRE–gene pair is annotated with higher number of Hi-C reads or is confirmed with Hi-C reads from more cell lines, it tends to have higher PCC (Figure 3D and E). As in these two conditions we would expect to observe a higher fraction of true DRE–target gene pairs, these results further validate that DRE and its target genes are strongly phylogenetic correlated.

Based on the above results, Hi-C annotated DRE–gene pairs with higher PCC or Hi-C read counts are more likely to be true DRE–target gene pairs. To determine the cutoffs of PCC and Hi-C read counts for selecting DRE–target gene pairs, we compute the repeatability of Hi-C annotated DRE–gene pairs between the replica cell lines at a series of cutoffs (PCC: 0.7, 0.8 and 0.9, and Hi-C read counts: 1, 2 and 3 and more).

**Figure 4.**
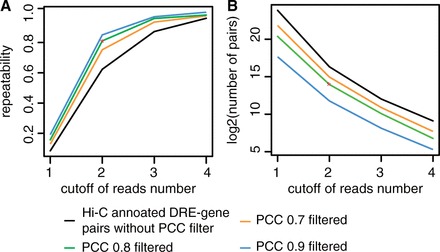
The repeatability of Hi-C annotated DRE–gene pairs between cell line replicates. (**A**) The repeatability of Hi-C annotated DRE–gene pairs between the two replicas of IMR90 cell line. (**B**) The number of Hi-C annotated DRE–gene pairs in IMR90 rep2 with different cutoffs.

Here, for the two replica of a given cell line (e.g. IMR90), we first choose the replica that have lower Hi-C reads at different read cutoffs, and identify the Hi-C annotated DRE–gene pairs with Hi-C reads above the read cutoff in this replica. The repeatability is then defined as the ratio of these Hi-C annotated DRE–gene pairs that can be confirmed in another replica without using the read cutoffs. A higher repeatability indicates a lower chance of observing DRE–gene pairs confirmed by noisy Hi-C data, and consequently a higher chance of identifying true DRE–target gene pairs. We find that the repeatability is significantly improved with the increase of PCC and the number of Hi-C reads (Figure 3A and Supplementary Figure S4). We choose the PCC cutoff to be >0.8 and the cutoff of Hi-C read counts to be >2 to predict DRE–target gene pairs, as they would not only produce a higher repeatability (0.81 in Figure 4A) but also result in a relatively large number of DRE–gene pairs (Figure 4B). In addition, for those DRE–gene pairs that have a PCC >0.8 and are confirmed by only one Hi-C read in one replica cell line, if they can be confirmed by the Hi-C data in another replica cell line, we also consider them as DRE–target gene pairs. Finally, we combine the predictions from the two cell lines, and obtain 260 897 predicted DRE–target gene pairs among which 48.4% are present in both cell lines (Supplementary Figure. S3B).

### Validation of predicted DRE–target gene relationships

The predicted DRE–target gene pairs correspond to 5344 genes and 104 636 DREs. A gene is usually regulated by multiple DREs (Supplementary Figure S5A). Nearly half of genes (47%) are regulated by nine or more DREs, and >6.6% of them are regulated by >200 DREs. As there are a large number of DREs and DREs regulating the same gene tend to be located approximately to each other (Supplementary Figure S6), we cluster DREs that are within 5 kb distance to each other. The 104 636 DREs are clustered into 44 760 DRE clusters, and genes regulated by a DRE within a DRE cluster are considered to be regulated by the corresponding DRE cluster.

**Figure 5.**
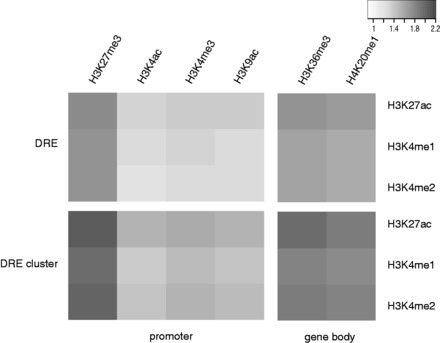
The enrichment of histone modification patterns among predicted DRE–target gene pairs. The observed-to-expected ratios of the combination of the histone modifications at DRE and at gene promoter or gene body among predicted DRE–target gene pairs (**A**) or among predicted DRE cluster–target gene pairs (**B**) are shown in the black and white heatmaps. The intensity of the black color corresponds to the observed-to-expected ratio. The analysis is performed on IMR90 cell line. The result of h1ESC is similar, and is shown in Supplementary Figure S7.

To validate the predicted DRE–target gene relationships, we consider two approaches. In the first approach, given that there are specific active/repressive histone markers at DREs or genes, we investigate whether the combination of histone markers of DREs and of genes are significantly enriched among the predicted DRE–target gene pairs. The selected histone markers for DREs are H3K4me1/2 (36) and H3K27ac (37). The selected active histone markers for genes are H3K4me3, H3K4/9ac at promoter and H3K36me3, H4K20me1 at gene body (38), and the repressive marker is H3K27me3 (38) at promoter. We download the ChIP-seq data of these histone markers on the two cell lines (25). Then, for a given combination of histone markers such as H3K4me1 at DRE and H3K36me3 at gene body, we calculate the observed-to-expected ratio of predicted DRE–target gene pairs with the two markers. Here the expected ratio is calculated by simply multiplying the ratio of DREs with one histone marker and the ratio of genes with another histone marker. For all the combination of histone markers investigated, the observed-to-expected ratios are higher than 1 (the *P*-values are all significant according to chi-square test) (Figure 5A). In addition, in general, for active DREs, our results show that the target genes are more likely to have H3K36me3 and H4K20me1 at gene body and H3K27me3 at promoter than the other histone markers. Interestingly, H3K27me3 is a repressive maker, indicating that a significant fraction of DREs may act as repressors. We also repeat the same analysis for predicted DRE cluster–target gene relationships, and find that all above combinations of are even more enriched among DRE cluster–target gene pairs than among DRE–target gene pairs (Figure 5B). The enrichment of the combination of histone markers specific for DREs and genes among our predicted DREs–target gene pairs therefore indirectly suggest a regulatory relationship between the predicted DREs and target genes.

In another approach, given that the regulation of DRE on target genes requires the mediation of certain TFs, such as EP300 (39), we investigate whether the expression of the predicted target genes of DREs is affected upon the knockout of the TF. Because knockout of EP300 is lethal (40) while the binding of EP300 requires CREB (39), we download the gene expression data of CREB knockout K562 cell line (41). Here, the reason why K562 cell line is used is because there are no available data for CREB knockout H1 hES or IMR90 cell lines. Meanwhile, we download the ChIP-seq data of EP300 for K562 cell line (25) to identify DREs that bind EP300. Next, we identify those genes that are significantly differential expressed in between normal and CREB knockout cell lines (*P* < 0.05, and with a fold change >1.5 or <0.7). Among those genes that have been predicted to be regulated by DREs, ∼15.9% are differentially expressed. In contrast, for those genes whose corresponding predicted DREs are bound by EP300, this percentage is 21.8%. The difference is statistically significant (*P* = 1.3e–4 according to Fisher’s exact test). As CREB binds not only DREs but also gene promoters, we exclude all CREB binding genes and redo the analysis. The percentage is 21.7%, which is still significantly higher than background (*P* = 2.5e–3). For the predicted target genes of DRE clusters, this percentage is 21.1 and 21.3% before and after the filtering of CREB binding genes. Both percentages are statistically significant than background (*P* = 6.2e–5 and 8.6e–5, respectively). Thus, our results show that the expression of the predicted target genes is more likely to be affected than that of random genes when the corresponding DREs are affected, which is consistent with our predictions.

### Functional analysis of predicted DRE cluster–target gene pairs

We investigate the function of those genes regulated by large number of DRE clusters. *DLG2*, *TMPRSS3* and *PDE4D* are the top three genes regulated by the most number of DREs. *DLG2* is located at 11q14.1, and is regulated by 729 DRE clusters in which 532 are located at chr11. It encodes a postsynaptic density protein, which is required for perception of chronic pain (25). The dysregulation of it in hippocampus is found to be related with major depressive disorder (42). *TMPRSS3* is located at 21q22.3, and is regulated by 692 DRE clusters in which 102 are located in chr21. This gene encodes a transmembrane protease, and is found to be differently expressed in tumors (43). Defects in *TMPRSS3* can cause deafness autosomal recessive type 8 (44). *PDE4D* is located at chromosome 5q12.1, and is regulated by 673 DRE clusters in which 523 are located at chr5. It encodes a cAMP hydrolase. Its genetic variation causes acrodysostosis type 2 (45), and may be related with risk of stroke (46). We perform further function enrichment analysis on the top 5% genes (271 genes) ranked by the number of DREs. Interestingly, we find that these genes are significantly enriched with essential functions, such as nervous system development (GO:0007399) and cell adhesion (GO:0007155) (Supplementary Table S2). In comparison, those genes regulated by only one DRE cluster are not enriched with any GO terms (adjusted *P* > 0.05).

**Figure 6.**
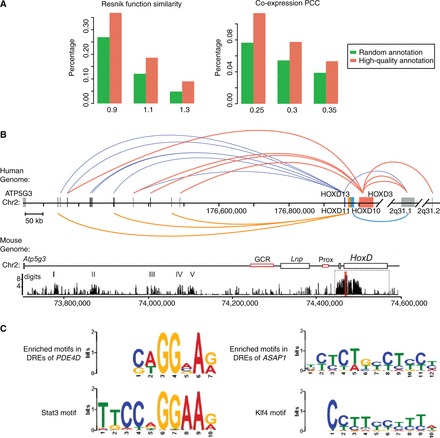
Functional analysis of predicted DRE–target gene pairs. (**A**) The proportion of DRE cluster whose mean Resnik function similarity score (left) or mean co-expression PCC (right) is above different cutoffs. The background proportions are obtained by randomly selecting the same number of genes related to a DRE cluster. (**B**) An example of literature validated DRE–target gene relationships. The upper subfigure refers to our predictions, while the lower subfigure is adopted from a previous study (47). DRE clusters are shown in black bars, while *HOXD* genes are shown in colored bars. DRE clusters regulating a *HoxD* gene is connected to the gene by curves with the same color of the gene. (**C**) Top left and right are the predicted motifs enriched in the DREs of *PDE4D and ASAP1*, respectively. Bottom left and right are the binding site motifs of STAT3 and KLF4, respectively.

Most DRE clusters regulate only one or two genes. But there are also a large number of DRE cluster regulating more genes. For example, over 1000 DRE clusters are found to regulate ≥10 genes (Supplementary Figure S5B). For genes regulated by the same DRE cluster, it is likely that those genes may be functionally related because of the common regulatory mechanism. To investigate this possibility, for each DRE cluster regulating three or more genes, we compute a mean Resnik functional similarity score (32) between genes regulated by the cluster. The Resnik score is computed using the GO annotations of two genes; with a higher Resnik score indicating a higher functional association (a random pair of genes would have a Resnik score of 0.73). As a control experiment, we randomly select the same number of genes from the genome, and calculate their mean Resnik score. Then, we compute the proportion of DRE clusters whose mean Resnik score is above a given cutoff, and compare it with the proportion obtained from the control experiments. We find that at a given Resnik score cutoff, the proportion of DRE clusters is significantly higher than that obtained from the control experiments (*P* < 2.2e–16) (Figure 6A), indicating that genes regulated by the same DRE cluster tend to be functionally related. We also investigate whether genes regulated by the same DRE cluster tend to be co-expressed. We download a gene expression data set that includes the expression profiles of 79 human tissues (33), and calculate the co-expression PCC for every pair of genes using the gene expression profiles. Then, we repeat the above experiments by replacing Resnik functional similarity score with co-expression PCC, and find that similar to the experiment of functional relatedness, at a given co-expression PCC cutoff, the proportion of DRE clusters is significantly higher than that obtained from the control experiment (*P* < 2.2e–16) (Figure 6A), indicating that genes regulated by the same DRE cluster tend to be co-expressed.

However, genes regulated by the same DRE cluster tend to be located near to each other, and those genes are more likely to be co-expressed and functionally related (Supplementary Figure S7), which may cause some bias to the above results. We then filter out the gene pairs that are within 500 kb to each other, and repeat the above analysis. The conclusions still hold true, though the result of co-expression PCC is not as significant as before (Supplementary Figure S8). Thus, we conclude distal genes regulated by the same DRE cluster also tend to be functionally related and co-expressed.

### Literature validation and application of predicted DRE–target gene pairs

In this study, we focus on predicting distal DRE–target gene pairs that are either located >500 kb distance from each other or from different chromosomes. Most current studies on DRE regulation focus on DRE and genes that are relatively close to each other in genome, making it difficult to find direct literature evidence to validate our predictions. We then search for experimentally validated DRE–target gene relationships in other genomes, and find one example that supports our predictions. In this example, a regulatory archipelago located in a 600 kb-long gene desert between *Atp5g3* and *HoxD13* in mouse genome was found to regulate the expression of *HoxD* genes (48). *HoxD* genes perform essential functions in many development process, and defects on *HoxD* genes would cause various limb malformation (44). We map this mouse regulatory archipelago to the human genome, and find it contains 21 DRE clusters in which 9 regulate *HOXD* genes according to our predictions. These *HOXD* genes include *HOXD13* (regulated by six DRE clusters), *HOXD11* (regulated by three DRE clusters) and *HOXD3* (regulated by four DRE clusters) (Figure 6B). In addition, we find several DRE clusters downstream to the *HOXD* gene clusters that regulate *HOXD* genes. Therefore, our predictions provide a great resource for experimental biologists to derive novel hypothesis and design new experiments to study distal gene regulations.

Because genes regulated by the same DRE cluster tend to be functionally related, it is possible to use the predictions to infer the function of unknown genes. To test this possibility, we perform GO enrichment analysis without using GO IEA evidence (computational predictions) on 56 DRE clusters regulating >23 genes. Among them, 17 have significantly enriched GO terms (Supplementary Table S3). Then, we investigate whether any genes inside these clusters are annotated with the enriched GO terms by IEA evidence. In one example, the DRE cluster 22833 (the ID of the DRE cluster defined in this study, see Supplementary File for details) (chr3:34039485-34072215) is enriched with the function of brain development (GO:0007420). Among the genes regulated by the cluster, *RARB* and *EXT1* are both annotated with this GO term by IEA. In another example, the DRE cluster 29008 (chr4:190563175-190584645) is enriched with the function of cell–cell adhesion (GO:0016337). *PCDH7* is regulated by this cluster, and is annotated with the same GO term by IEA. Therefore, function of unknown genes may be inferred based on the patterns of DRE regulation.

The predicted DRE–target gene relationships also offer an opportunity to investigate the regulatory mechanisms between DRE and target gene. PDE4D, an important cAMP-specific phosphodiesterase (44), is predicted to be regulated by 1287 DREs. Motif enrichment analysis by Multiple EM for Motif Elicitation (MEME) (motif length is set to be 5–20 bp with default parameter setting) (48) on these DREs reveals that a 7 bp motif is enriched in 880 DREs (E-value = 7.0e-38). This motif is found to be highly similar to the STAT3 motif by TOMTOM (49) against the JASPAR database (core 2009) (Figure 6C). STAT3 is a transcription activator that mediates cellular responses to growth factors (44). Previous study has shown that the PI3K−PDE3B−cAMP pathway is interacting with the Jak2−Stat3 pathways (50). Because both PDE4D and PDE3B are members of cAMP phosphodiesterase family and PDE4D may share common inhibitor with *PDE3B* (51), our analysis suggests that STAT3 binding to the DRE may play an important role in regulating the expression of *PDE4D*. In another example, out of 1030 DREs predicted to regulate *ASAP1*, 1028 are found to contain a 12-bp motif (E-value = 5.5e–102) that is similar to the KLF4 motif (Figure 6C). ASAP1 is involved in the differentiation of fibroblasts into adipocytes (44), and was previously named differentiation-enhancing factor 1. KLF4 is a stem-cell TF interacting with CREB binding protein (52). A previous study showed that KLF4 regulates the expression of *FAK*, an intracellular tyrosine kinase related to neovascularization in endothelial cells (53). Because ASAP1 is interacting with FAK (52), this suggests that KLF4 may regulate the expression of *ASAP1* through binding to its DREs.

Recent analysis on disease-associated noncoding SNPs has revealed that many of them are located within regulatory elements (10). Here we show with a couple of examples that our predictions can be used to explain the casual roles of disease-associated regulatory SNPs. SNP rs11610206 is associated with Alzheimer’s disease (54). This SNP is located within a noncoding region, and the nearest coding gene to this SNP is *FAM113B* whose function is unknown. Thus, the exact reason why this SNP is disease associated is unclear. We find this SNP is located within the DRE cluster 7987 (chr12:47637135-47642135) whose target gene is *VDR*. *VDR* is located 600 kb downstream to the DRE cluster. It encodes the Vitamin D3 receptor that is a TF regulating many hormone sensitive genes. The suppression of *VDR* has been found to be related with Alzheimer’s disease in several studies (55–57). Therefore, it is likely the reason why SNP rs11610206 is associated with Alzheimer’s disease is because this SNP influences the function of the DRE, which then affects the expression of *VDR* and leads to Alzheimer’s disease. SNP rs7578326 is another example. It is associated with type II diabetes according to a genome-wide association study (58). This SNP is located within the intron of a noncoding RNA, BC017935, whose function is unknown, making it difficult to explain the causal role of this SNP. We find this SNP is located within the DRE cluster 20449 (chr2:227013575-227022415), which targets a 500 kb downstream gene that encodes IRS1 (insulin receptor substrate 1). Because IRS1 signaling is essential for glucose homeostasis in liver (59), and is related with the insulin sensitivity (60) and resistance (61), it is likely that this SNP may affect the function of DRE, which may impact the expression of IRS1 and contribute to type II diabetes. Given the increasing awareness of disease-associated noncoding SNPs, our predictions provide a valuable resource for explaining the causal roles of disease-associated SNPs in DRE region.

## DISCUSSION

Defining the target genes of DREs, including enhancers, repressors and insulators, is crucial to our understanding of the function of DREs and the mechanisms of long-range regulation. In this study we develop a method that combines phylogenetic correlation with Hi-C read counts to predict distal DREs–target gene pairs that are located >500 kb from each other or from different chromosomes. Analysis of the predicted DRE–target gene pairs reveal that genes regulated by a large number of DREs tend to have essential functions, and genes regulated by the same DREs tend to be functionally related and co-expressed. We consider the predicted DRE–target gene pairs are of high quality, as they not only are captured by Hi-C but also show strong phylogenetic correlation. In addition, these predictions are validated by the histone modification patterns and the differential gene expression pattern in a CREB knockout cell line. However, there may still exist false positives, which can be attributed to the following reasons. The read cutoff is set at two reads, which is not a stringent cutoff, and may result in false positives. On the other hand, phylogenetic correlation is not a sensitive measure, and can be affected by the nearby genomic context of the gene or DREs under consideration, which may cause the inclusion of false positives as well. As for false negatives, first there is always a tradeoff of reducing false positives. Second, in this study we only analyze intergenic DREs, while DHS inside a gene may also act as DRE of other genes. Third, we focus only on distal DRE–target gene relationships. Fourth, not all DRE–target genes are phylogenetic correlated, especially for those newly evolved DRE–target gene relationships. Finally, DRE–target gene relationships may be cell-type specific, and our predictions are based only on two cell lines. Nevertheless, our predictions are of great value for experimental biologists to design new experiments to study the mechanisms of long-range regulation. In addition, these predictions can be explored to infer the function of uncharacterized genes. Furthermore, given the increasing awareness of the importance of noncoding SNPs with disease association from recent genome-wide association studies (10,62,63), the predicted target genes of DREs are of importance for elucidating the causal roles of those SNPs that are located in DREs.

## SUPPLMENTARY DATA

Supplementary Data are available at NAR Online.

## FUNDING

Funding for open access charge: National Basic Research Program of China [2012CB316505, 2010CB529505]; the National Natural Science Foundation of China [91231116, 31071113, 30971643]; the Specialized Research Fund for the Doctoral Program of Higher Education of China [20120071110018]; the Innovation Program of Shanghai Municipal Education Commission [13ZZ006]; FDUROP, Fudan’s Undergraduate Research Opportunity Program (to Y.Z.).

*Conflict of interest statement*. None declared.

## Supplementary Material

Supplementary Data

## References

[1] Mossing MC, Record MT (1986). Upstream operators enhance repression of the lac promoter. Science.

[2] Ong CT, Corces VG (2011). Enhancer function: new insights into the regulation of tissue-specific gene expression. Nat. Rev. Genet..

[3] Chan KK, Zhang J, Chia NY, Chan YS, Sim HS, Tan KS, Oh SK, Ng HH, Choo AB (2009). KLF4 and PBX1 directly regulate NANOG expression in human embryonic stem cells. Stem Cells.

[4] Yeom YI, Fuhrmann G, Ovitt CE, Brehm A, Ohbo K, Gross M, Hubner K, Scholer H (1996). Germline regulatory element of Oct-4 specific for the totipotent cycle of embryonal cells. Development.

[5] Pregizer S, Mortlock DP (2009). Control of BMP gene expression by long-range regulatory elements. Cytokine Growth Factor Rev..

[6] Kleinjan DA, Seawright A, Schedl A, Quinlan RA, Danes S, van Heyningen V (2001). Aniridia-associated translocations, DNase hypersensitivity, sequence comparison and transgenic analysis redefine the functional domain of PAX6. Hum. Mol. Genet..

[7] Emison ES, McCallion AS, Kashuk CS, Bush RT, Grice E, Lin S, Portnoy ME, Cutler DJ, Green ED, Chakravarti A (2005). A common sex-dependent mutation in a RET enhancer underlies Hirschsprung disease risk. Nature.

[8] Lettice LA, Heaney SJ, Purdie LA, Li L, de Beer P, Oostra BA, Goode D, Elgar G, Hill RE, de Graaff E (2003). A long-range Shh enhancer regulates expression in the developing limb and fin and is associated with preaxial polydactyly. Hum. Mol. Genet..

[9] de Kok YJ, Merkx GF, van der Maarel SM, Huber I, Malcolm S, Ropers HH, Cremers FP (1995). A duplication/paracentric inversion associated with familial X-linked deafness (DFN3) suggests the presence of a regulatory element more than 400 kb upstream of the POU3F4 gene. Hum. Mol. Genet..

[10] Noonan JP, McCallion AS (2010). Genomics of long-range regulatory elements. Annual review of genomics and human genetics.

[11] Aparicio S, Morrison A, Gould A, Gilthorpe J, Chaudhuri C, Rigby P, Krumlauf R, Brenner S (1995). Detecting conserved regulatory elements with the model genome of the Japanese puffer fish, Fugu rubripes. Proc. Natl Acad. Sci. USA.

[12] Coy S, Caamano JH, Carvajal J, Cleary ML, Borycki AG (2011). A novel Gli3 enhancer controls the Gli3 spatiotemporal expression pattern through a TALE homeodomain protein binding site. Mol. Cell. Biol..

[13] Vokes SA, Ji H, Wong WH, McMahon AP (2008). A genome-scale analysis of the cis-regulatory circuitry underlying sonic hedgehog-mediated patterning of the mammalian limb. Genes Dev..

[14] Dorsett D (1999). Distant liaisons: long-range enhancer-promoter interactions in Drosophila. Curr. Opin. Genet. Dev..

[15] Ferretti E, Cambronero F, Tumpel S, Longobardi E, Wiedemann LM, Blasi F, Krumlauf R (2005). Hoxb1 enhancer and control of rhombomere 4 expression: complex interplay between PREP1-PBX1-HOXB1 binding sites. Mol. Cell. Biol..

[16] Rebeiz M, Reeves NL, Posakony JW (2002). SCORE: a computational approach to the identification of cis-regulatory modules and target genes in whole-genome sequence data. Site clustering over random expectation. Proc. Natl Acad. Sci. USA.

[17] Thurman RE, Rynes E, Humbert R, Vierstra J, Maurano MT, Haugen E, Sheffield NC, Stergachis AB, Wang H, Vernot B (2012). The accessible chromatin landscape of the human genome. Nature.

[18] Dekker J, Rippe K, Dekker M, Kleckner N (2002). Capturing chromosome conformation. Science.

[19] Lieberman-Aiden E, van Berkum NL, Williams L, Imakaev M, Ragoczy T, Telling A, Amit I, Lajoie BR, Sabo PJ, Dorschner MO (2009). Comprehensive mapping of long-range interactions reveals folding principles of the human genome. Science.

[20] Fullwood MJ, Liu MH, Pan YF, Liu J, Xu H, Mohamed YB, Orlov YL, Velkov S, Ho A, Mei PH (2009). An oestrogen-receptor-alpha-bound human chromatin interactome. Nature.

[21] Gross DS, Garrard WT (1988). Nuclease hypersensitive sites in chromatin. Annu. Rev. Biochem..

[22] Crawford GE, Davis S, Scacheri PC, Renaud G, Halawi MJ, Erdos MR, Green R, Meltzer PS, Wolfsberg TG, Collins FS (2006). DNase-chip: a high-resolution method to identify DNase I hypersensitive sites using tiled microarrays. Nat. Methods.

[23] Song L, Crawford GE (2010). DNase-seq: a high-resolution technique for mapping active gene regulatory elements across the genome from mammalian cells. Cold Spring Harb. Protoc..

[24] Dixon JR, Selvaraj S, Yue F, Kim A, Li Y, Shen Y, Hu M, Liu JS, Ren B (2012). Topological domains in mammalian genomes identified by analysis of chromatin interactions. Nature.

[25] Birney E, Stamatoyannopoulos JA, Dutta A, Guigo R, Gingeras TR, Margulies EH, Weng Z, Snyder M, Dermitzakis ET, Thurman RE (2007). Identification and analysis of functional elements in 1% of the human genome by the ENCODE pilot project. Nature.

[26] Kent WJ, Sugnet CW, Furey TS, Roskin KM, Pringle TH, Zahler AM, Haussler D (2002). The human genome browser at UCSC. Genome Res..

[27] Flicek P, Amode MR, Barrell D, Beal K, Brent S, Chen Y, Clapham P, Coates G, Fairley S, Fitzgerald S (2011). Ensembl 2011. Nucleic Acids Res..

[28] Bernstein BE, Stamatoyannopoulos JA, Costello JF, Ren B, Milosavljevic A, Meissner A, Kellis M, Marra MA, Beaudet AL, Ecker JR (2010). The NIH roadmap epigenomics mapping consortium. Nat. Biotechnol..

[29] Zhou Y, Lu Y, Tian W (2012). Epigenetic features are significantly associated with alternative splicing. BMC Genomics.

[30] Zhang Y, Liu T, Meyer CA, Eeckhoute J, Johnson DS, Bernstein BE, Nusbaum C, Myers RM, Brown M, Li W (2008). Model-based analysis of ChIP-Seq (MACS). Genome Biol..

[31] Berriz GF, King OD, Bryant B, Sander C, Roth FP (2003). Characterizing gene sets with FuncAssociate. Bioinformatics.

[32] Resnik P (1995). Using information content to evaluate semantic similarity in a taxonomy. Arxiv preprint cmp-lg/9511007.

[33] Su AI, Wiltshire T, Batalov S, Lapp H, Ching KA, Block D, Zhang J, Soden R, Hayakawa M, Kreiman G (2004). A gene atlas of the mouse and human protein-encoding transcriptomes. Proc. Natl Acad. Sci. USA.

[34] Handoko L, Xu H, Li G, Ngan CY, Chew E, Schnapp M, Lee CW, Ye C, Ping JL, Mulawadi F (2011). CTCF-mediated functional chromatin interactome in pluripotent cells. Nat. Genet..

[35] Li G, Ruan X, Auerbach RK, Sandhu KS, Zheng M, Wang P, Poh HM, Goh Y, Lim J, Zhang J (2012). Extensive promoter-centered chromatin interactions provide a topological basis for transcription regulation. Cell.

[36] Heintzman ND, Stuart RK, Hon G, Fu Y, Ching CW, Hawkins RD, Barrera LO, Van Calcar S, Qu C, Ching KA (2007). Distinct and predictive chromatin signatures of transcriptional promoters and enhancers in the human genome. Nat. Genet..

[37] Creyghton MP, Cheng AW, Welstead GG, Kooistra T, Carey BW, Steine EJ, Hanna J, Lodato MA, Frampton GM, Sharp PA (2010). Histone H3K27ac separates active from poised enhancers and predicts developmental state. Proc. Natl Acad. Sci. USA.

[38] Dunham I, Birney E, Lajoie BR, Sanyal A, Dong X, Greven M, Lin X, Wang J, Whitfield TW, Zhuang J (2012). An integrated encyclopedia of DNA elements in the human genome. Nature.

[39] Vo N, Goodman RH (2001). CREB-binding protein and p300 in transcriptional regulation. J. Biol. Chem..

[40] Pasqualucci L, Dominguez-Sola D, Chiarenza A, Fabbri G, Grunn A, Trifonov V, Kasper LH, Lerach S, Tang H, Ma J (2011). Inactivating mutations of acetyltransferase genes in B-cell lymphoma. Nature.

[41] Pellegrini M, Cheng JC, Voutila J, Judelson D, Taylor J, Nelson SF, Sakamoto KM (2008). Expression profile of CREB knockdown in myeloid leukemia cells. BMC Cancer.

[42] Duric V, Banasr M, Stockmeier CA, Simen AA, Newton SS, Overholser JC, Jurjus GJ, Dieter L, Duman RS (2013). Altered expression of synapse and glutamate related genes in post-mortem hippocampus of depressed subjects. Int. J. Neuropsychopharmacol..

[43] Wallrapp C, Hahnel S, Muller-Pillasch F, Burghardt B, Iwamura T, Ruthenburger M, Lerch MM, Adler G, Gress TM (2000). A novel transmembrane serine protease (TMPRSS3) overexpressed in pancreatic cancer. Cancer Res..

[44] UniProt Consortium (2010). The Universal Protein Resource (UniProt) in 2010. Nucleic Acids Res..

[45] Michot C, Le Goff C, Goldenberg A, Abhyankar A, Klein C, Kinning E, Guerrot AM, Flahaut P, Duncombe A, Baujat G (2012). Exome sequencing identifies PDE4D mutations as another cause of acrodysostosis. Am. J. Hum. Genet..

[46] Rosand J, Bayley N, Rost N, de Bakker PI (2006). Many hypotheses but no replication for the association between PDE4D and stroke. Nat. Genet..

[47] Sur IK, Hallikas O, Vaharautio A, Yan J, Turunen M, Enge M, Taipale M, Karhu A, Aaltonen LA, Taipale J (2012). Mice lacking a Myc enhancer that includes human SNP rs6983267 are resistant to intestinal tumors. Science.

[48] Montavon T, Soshnikova N, Mascrez B, Joye E, Thevenet L, Splinter E, de Laat W, Spitz F, Duboule D (2011). A regulatory archipelago controls Hox genes transcription in digits. Cell.

[49] Bailey TL, Boden M, Buske FA, Frith M, Grant CE, Clementi L, Ren J, Li WW, Noble WS (2009). MEME SUITE: tools for motif discovery and searching. Nucleic Acids Res..

[50] Zhao AZ, Huan J-N, Gupta S, Pal R, Sahu A (2002). A phosphatidylinositol 3-kinase–phosphodiesterase 3B–cyclic AMP pathway in hypothalamic action of leptin on feeding. Nat. Neurosci..

[51] Moon E, Lee R, Near R, Weintraub L, Wolda S, Lerner A (2002). Inhibition of PDE3B augments PDE4 inhibitor-induced apoptosis in a subset of patients with chronic lymphocytic leukemia. Clinical cancer research.

[52] Geiman DE, Ton-That H, Johnson JM, Yang VW (2000). Transactivation and growth suppression by the gut-enriched Krüppel-like factor (Krüppel-like factor 4) are dependent on acidic amino acid residues and protein–protein interaction. Nucleic Acids Res..

[53] Wary KK, Kohler EE, Chatterjee I (2012). Focal adhesion kinase regulation of neovascularization. Microvasc. Res..

[54] Yu JT, Mao CX, Zhang HW, Zhang Q, Wu ZC, Yu NN, Zhang N, Li Y, Tan L (2011). Genetic association of rs11610206 SNP on chromosome 12q13 with late-onset Alzheimer's disease in a Han Chinese population. Clin. Chim. Acta.

[55] Dursun E, Gezen-Ak D, Yilmazer S (2011). A novel perspective for Alzheimer's disease: vitamin D receptor suppression by amyloid-beta and preventing the amyloid-beta induced alterations by vitamin D in cortical neurons. J. Alzheimers Dis..

[56] Sutherland MK, Somerville MJ, Yoong LK, Bergeron C, Haussler MR, McLachlan DR (1992). Reduction Of vitamin-D hormone receptor mRNA levels in Alzheimer as compared to huntington hippocampus: correlation with calbindin-28k mRNA levels. Brain Res. Mol. Brain Res..

[57] Gezen-Ak D, Dursun E, Ertan T, Hanagasi H, Guervit H, Emre M, Eker E, Oztuerk M, Engin F, Yilmazer S (2007). Association between vitamin D receptor gene polymorphism and Alzheimer's disease. Tohoku J. Exp. Med..

[58] Bao XY, Peng B, Yang MS (2012). Replication study of novel risk variants in six genes with type 2 diabetes and related quantitative traits in the Han Chinese lean individuals. Mol. Biol. Rep..

[59] Dong XC, Park SM, Lin XY, Copps K, Yi XJ, White MF (2006). Irs1 and Irs2 signaling is essential for hepatic glucose homeostasis and systemic growth. J. Clin. Invest..

[60] Copps KD, Hancer NJ, Opare-Ado L, Qiu W, Walsh C, White MF (2010). Irs1 Serine 307 promotes insulin sensitivity in mice. Cell Metab..

[61] Danielsson A, Ost A, Lystedt E, Kjolhede P, Gustavsson J, Nystrom FH, Stralfors P (2005). Insulin resistance in human adipocytes occurs downstream of IRS1 after surgical cell isolation but at the level of phosphorylation of IRS1 in type 2 diabetes. FEBS J..

[62] Visel A, Rubin EM, Pennacchio LA (2009). Genomic views of distant-acting enhancers. Nature.

[63] Maurano MT, Humbert R, Rynes E, Thurman RE, Haugen E, Wang H, Reynolds AP, Sandstrom R, Qu H, Brody J (2012). Systematic localization of common disease-associated variation in regulatory DNA. Science.

