# Supplementary material for: Combining Hi-C data with phylogenetic correlation to predict the target genes of distal regulatory elements in human genome
Source: Nucleic Acids Res. 2013 Sep 3;41(22):10391–402. doi: 10.1093/nar/gkt785 (PMC3905853; doi:10.1093/nar/gkt785)
Supplement: Supplementary Data [file supp_41_22_10391__index.html]

Combining Hi-C data with phylogenetic correlation to predict the target genes of distal regulatory elements in human genome — Combining Hi-C data with phylogenetic correlation to predict the target genes of distal regulatory elements in human genome — Supplementary Data 

# Combining Hi-C data with phylogenetic correlation to predict the target genes of distal regulatory elements in human genome

## Supplementary Data

files

**Files in this Data Supplement:**

- Supplementary Data - zip file
