# Supplementary material for: Combining Hi-C data with phylogenetic correlation to predict the target genes of distal regulatory elements in human genome
Source: Nucleic Acids Res. 2013 Sep 3;41(22):10391–402. doi: 10.1093/nar/gkt785 (PMC3905853; doi:10.1093/nar/gkt785)

**Supplementary Figure 1. The distribution of Hi-C reads around DHS. A.** The distribution of DHSs around TSS. **B**. The distribution of Hi-C reads around RNA Pol II. **C**. The distribution of Hi-C reads around DHSs. The processing procedures are the same as in **Fig. 1**, except that in (**A)** we count the number of DHS instead of Hi-C reads.

**Supplementary Figure 2. The correlation between gene length and Hi-C read counts for Hi-C annotated DRE-gene pairs.** For a given DRE-gene pair, the number of associated Hi-C reads is counted by two measures: sum, the sum of Hi-C read counts mapped to all DHS inside the gene, and max, the maximum number of Hi-C reads mapped to a DHS inside the gene. The correlation between Hi-C read counts and gene length is computed separately for Hi-C annotated DRE-gene pairs in the four cell lines (H1 hESC_rep1 and H1 hESC_rep2 refer to the two replica of h1 ESC cell line, while IMR90_rep1 and IMR90_rep2 refer to the two replica of IMR90 cell line) using R function cor.test, with methods “pearson” for Pearson correlation coefficients (**A**) and “spearman” for Spearman correlation coefficient (**B**).

**Supplementary Figure 3. Venn diagram of Hi-C annotated DRE-gene pairs**. Venn diagram of Hi-C annotated DRE-gene pairs before (**A**) and after (**B**) the application of PCC and Hi-C read counts cutoff in H1 hESC and IMR90 cell lines. **M** and **K** refer to million and thousand pairs, respectively.

**Supplementary Figure 4. The repeatability of Hi-C annotated DRE-gene pairs in between the replicates of H1 hESC cell line. A.** The repeatability of Hi-C annotated DRE-gene pairs in between the replicates of H1 hESC cell line at different cutoffs of PCC and Hi-C read counts. **B.** The number of Hi-C annotated DRE-gene pairs in H1 hESC rep2 cell lines at different cutoffs of PCC and Hi-C read counts.

**Supplementary Figure 5. The degree distribution of the predicted DRE-target gene network before and after DRE clustering.** The degree of genes is defined as the number of DREs (or DRE clusters) predicted to regulate the gene, while the degree of DREs (or DRE cluster) is defined as the number of genes predicted to be regulated by the DRE (or DRE cluster). **A** and **C** are the degree of genes before and after the clustering of DREs, respectively. **B** and **D** are the degree of DREs before and after the clustering of DREs, respectively.

**Supplementary Figure 6. The distribution of the distance.** This figure shows the distribution of the distance between genes regulated by the same DRE cluster **(A)** and the distance between DREs regulating the same gene **(B).** The distances are calculated for the pairs in the same chromosome. Background refers to random gene or DRE pairs that are in the same chromosome.

**Supplementary Figure 7. The enrichment of histone modification patterns among predicted DRE-target gene pairs in H1 hESC.** The heatmaps are plotted in the same way as in Fig.5.

**Supplementary Figure 8. Functional analysis of genes located within 500kb to each other. (A)** and **(B)** show the proportion of within-500kb gene pairs whose Resnik functional similarity and co-expression PCC above different cutoffs, respectively.

**Supplementary Figure 9. Functional analysis of predicted DRE-target gene pairs after filtering closely located genes (within 500kb).** The proportion of DRE cluster whose mean Resnik function similarity score (left) or mean co-expression PCC (right) is above different cutoffs. The processing procedures are the same as in **Fig. 5B.**

# Supplemental Figure 1

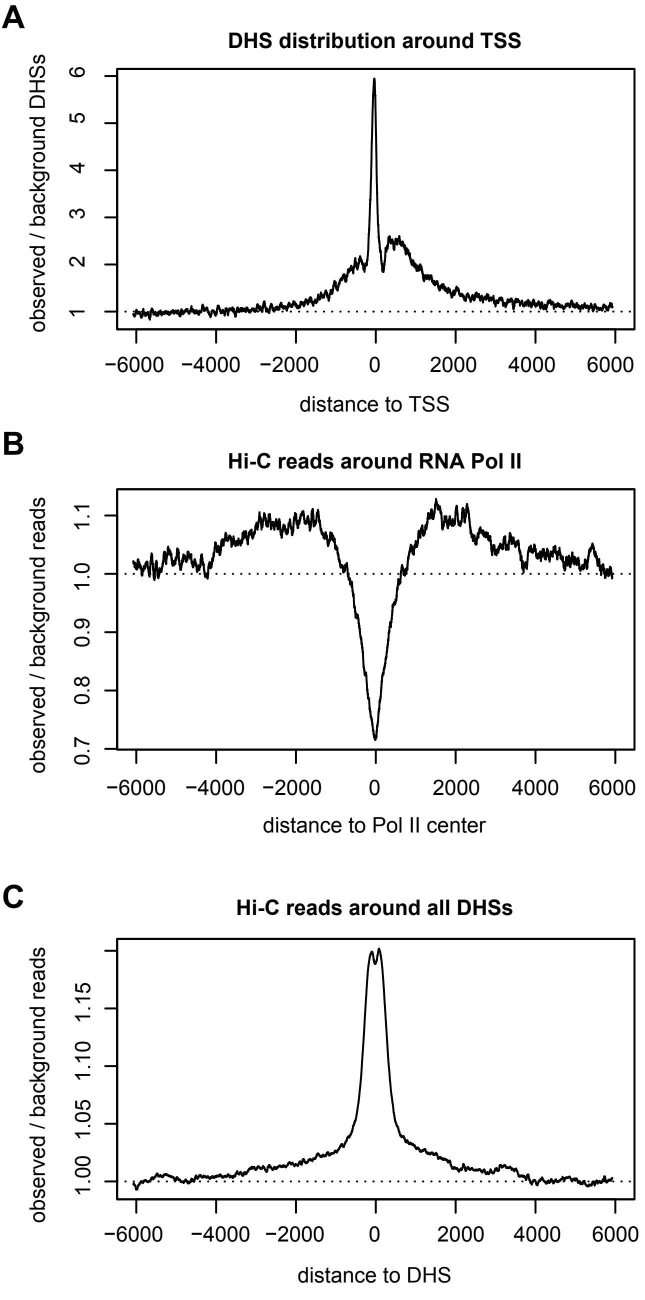

Supplemental Figure 2

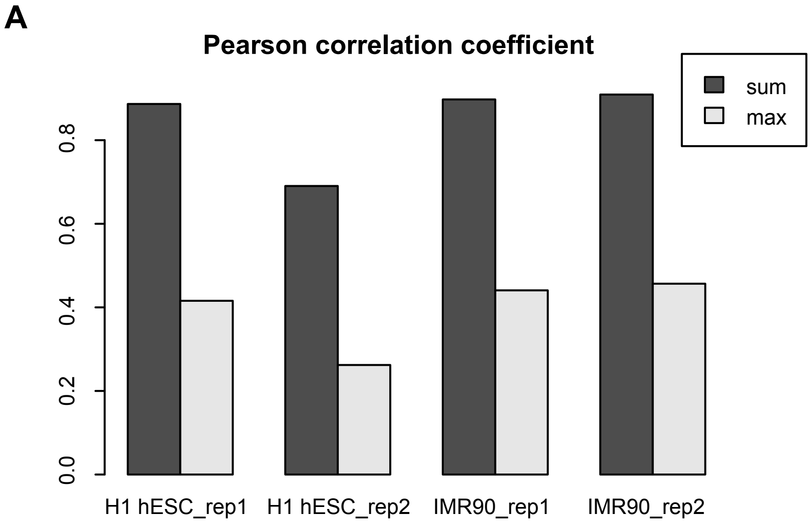


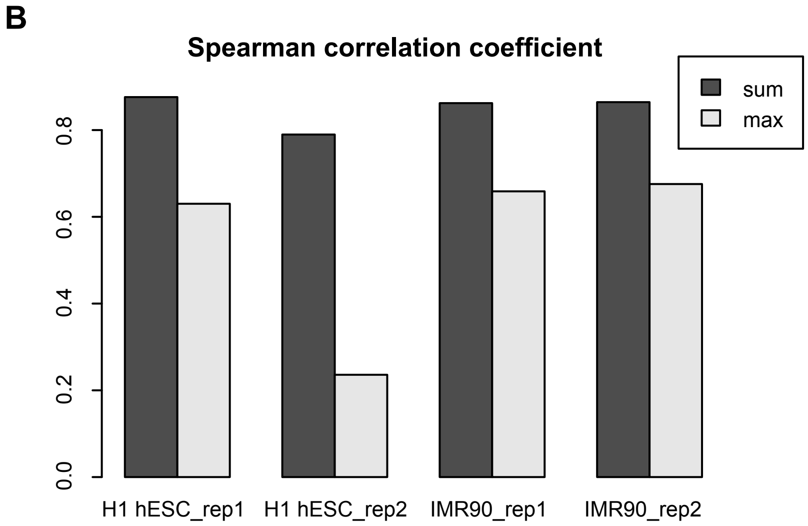

Supplemental Figure 3

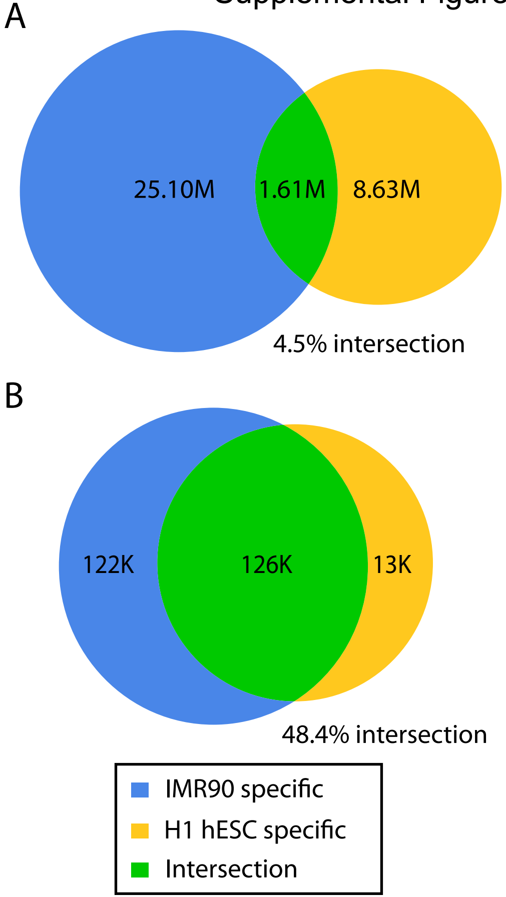

# Supplemental Figure 4

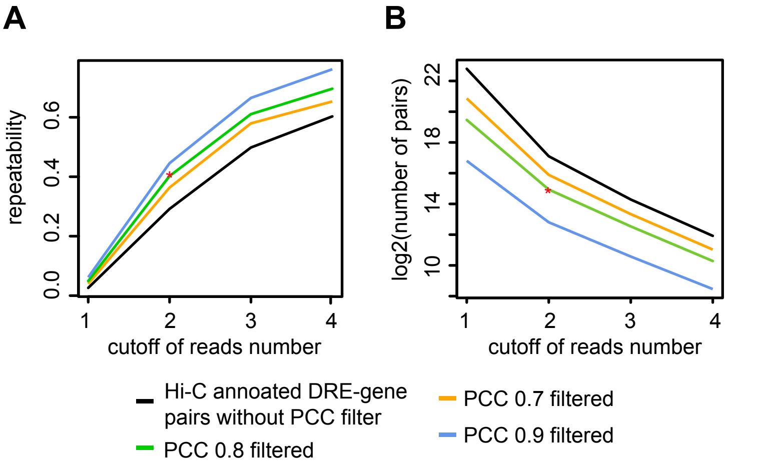

Supplemental Figure 5

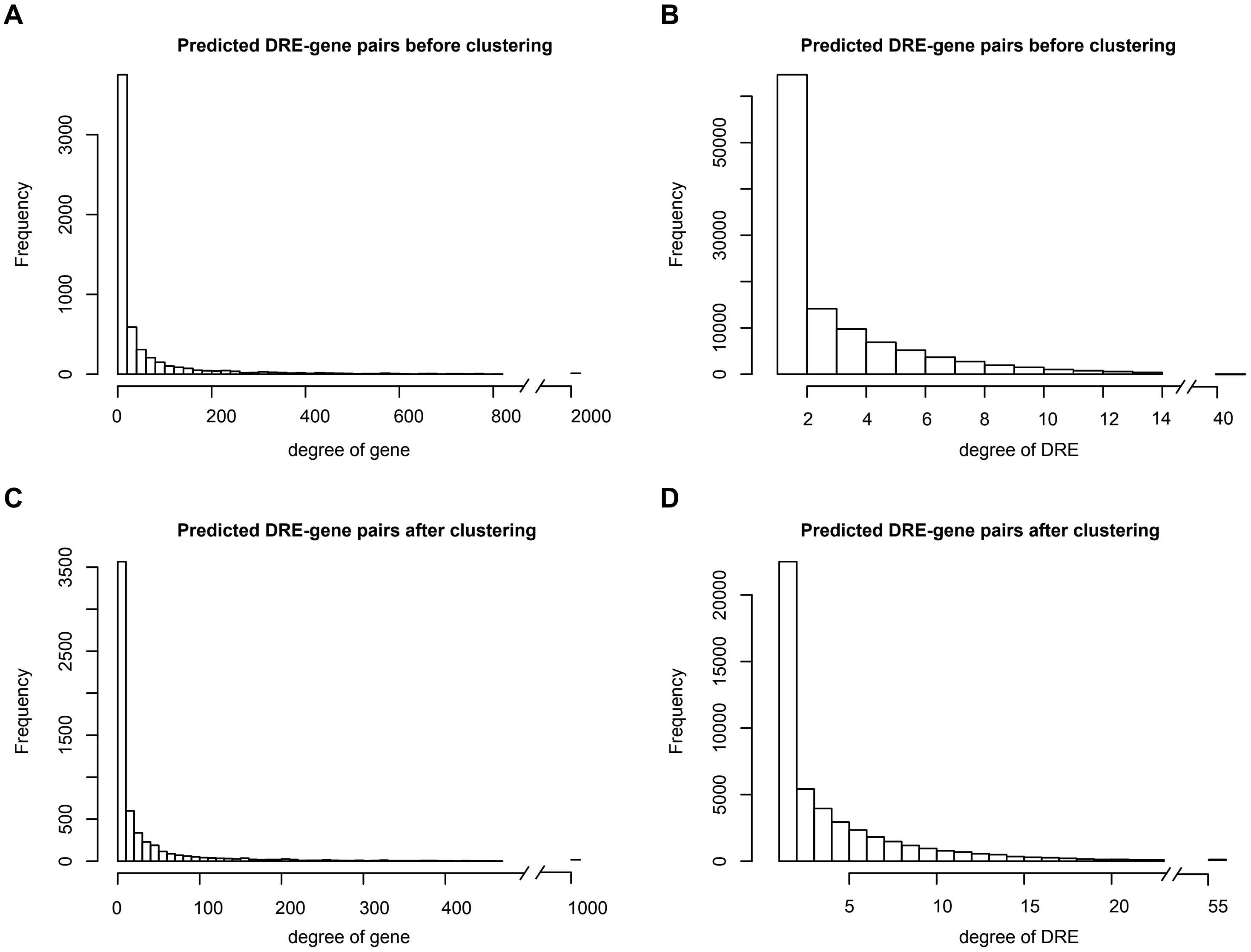

Supplemental Figure 6

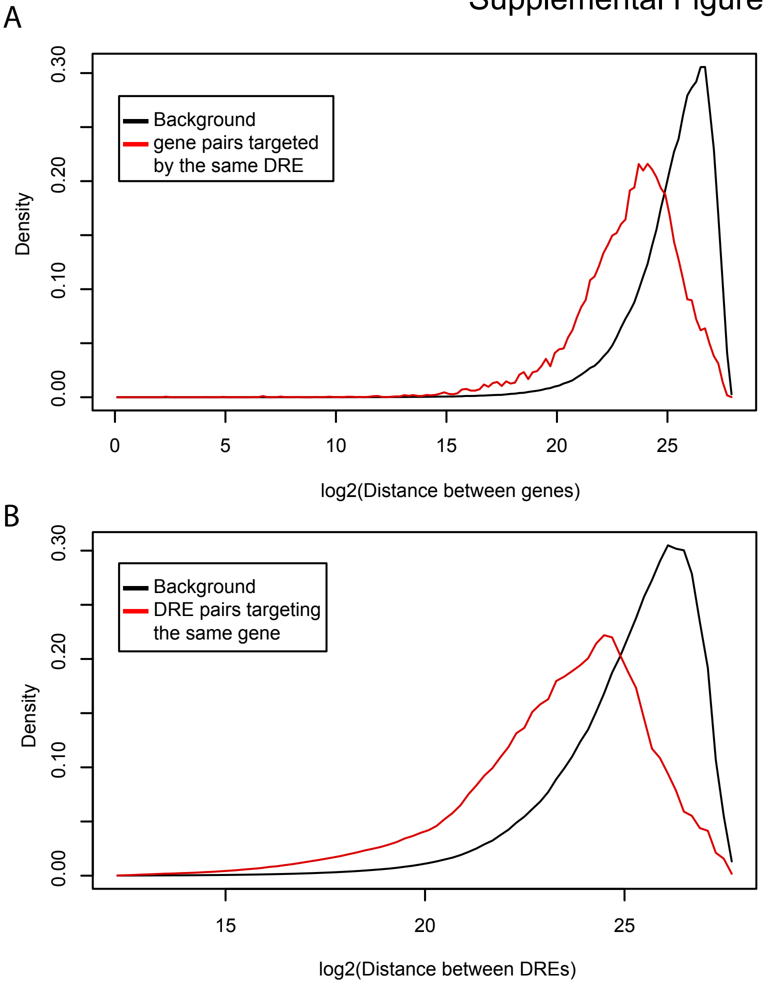

Supplemental Figure 7

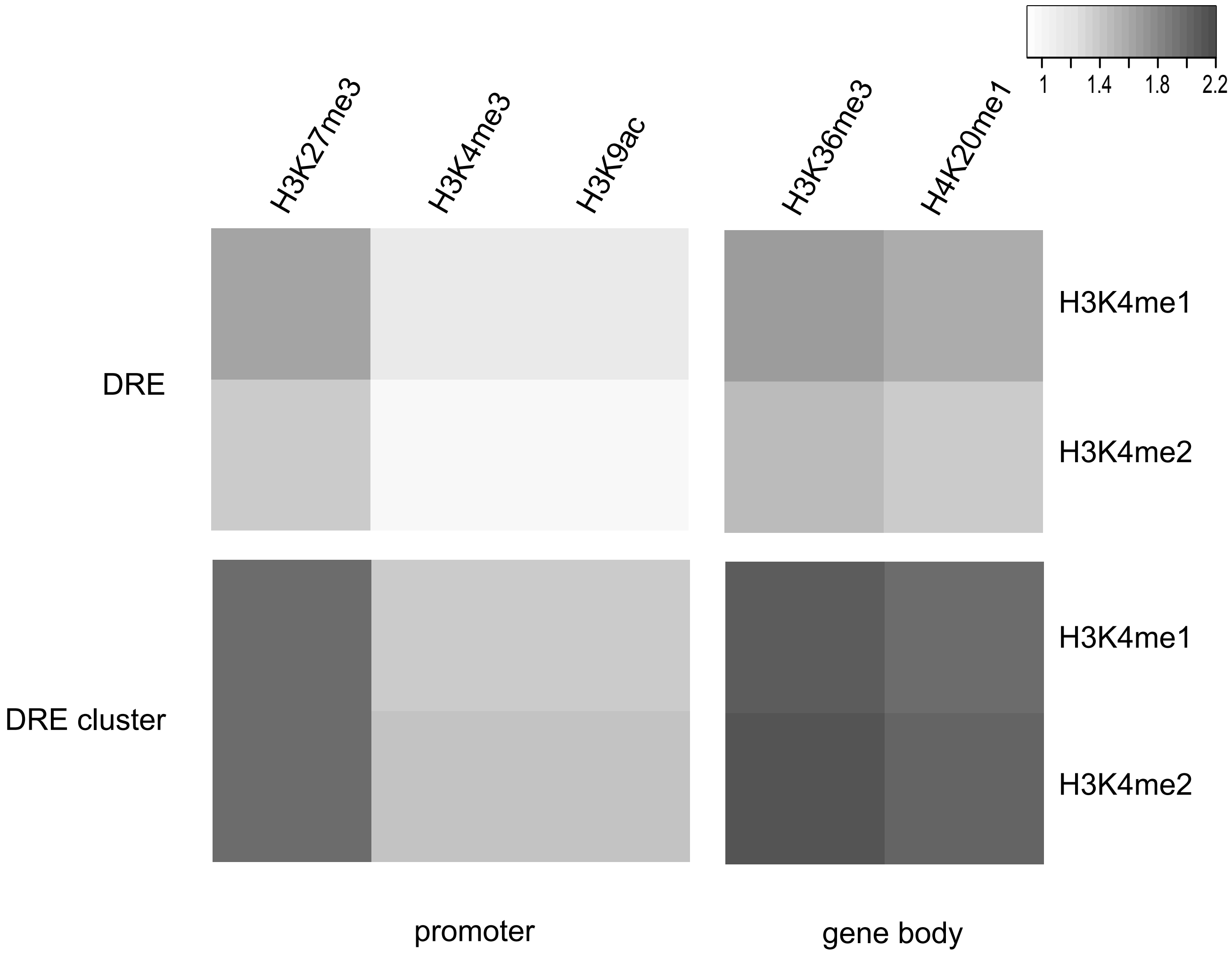

Supplemental Figure 8

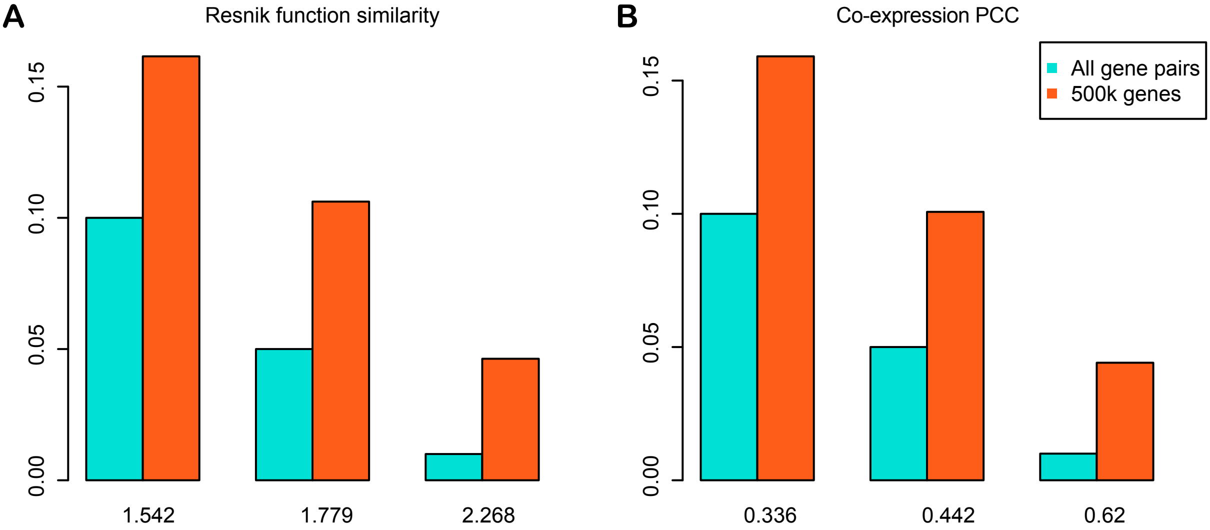

Supplemental Figure 9

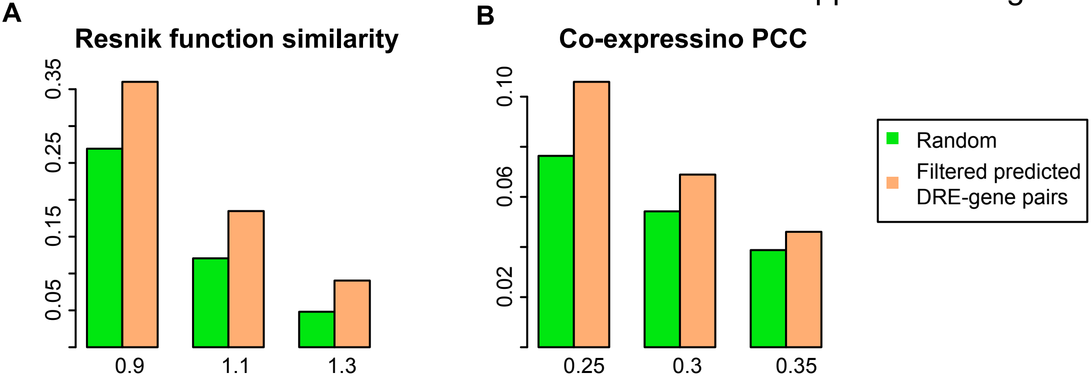

Supplement: Supplementary Data [file supp_gkt785_suppl_data.zip › nar-01376-n-2013-File010.pdf]
